# Electronic Cigarettes and Tobacco Product Cessation: A Survey of Healthcare Providers’ Opinions on Safety and Recommendation

**DOI:** 10.3390/healthcare12141410

**Published:** 2024-07-15

**Authors:** Surarong Chinwong, Thitichaya Penthinapong, Phitinan Tengcharoenphongthorn, Siroj Pingkaew, Khanchai Siriwattana, Arintaya Phrommintikul, Dujrudee Chinwong

**Affiliations:** 1 Department of Pharmaceutical Care, Faculty of Pharmacy, Chiang Mai University, Chiang Mai 50200, Thailand; surarong@gmail.com (S.C.); thitichaya.pen@cmu.ac.th (T.P.); phitinan_teng@hotmail.com (P.T.); pingkaew.siroj@gmail.com (S.P.); 2 Research Center for Innovation in Analytical Science and Technology for Biodiversity-Based Economic and Society (I-ANALY-S-T_B.BES-CMU), Chiang Mai University, Chiang Mai 50200, Thailand; 3 Division of Medicine, Nakornping Hospital, Chiang Mai 50180, Thailand; kancsiri@gmail.com; 4 Department of Internal Medicine, Faculty of Medicine, Chiang Mai University, Chiang Mai 50200, Thailand; arintaya.p@cmu.ac.th

**Keywords:** e-cigarettes, electronic cigarettes, smoking, tobacco control, healthcare providers, Thailand

## Abstract

*Background*: This study aimed to: (1) assess healthcare providers’ opinions on e-cigarette safety and compare them across professions; (2) evaluate providers’ recommendations for e-cigarettes as a tobacco product cessation tool and identify any associations with their safety perceptions. *Methods*: A self-administered questionnaire was completed by 760 healthcare professionals (January–March 2020). The survey included eight opinions on e-cigarette safety with five Likert-scale answers and a question on recommending them for tobacco product cessation. *Results*: Among 760 healthcare providers (173 physicians/dentists, 69 pharmacists, and 518 nurses), only 20% reported prior experience with tobacco product cessation counseling. Providers expressed uncertainty regarding e-cigarette safety (mean ± SD: 2.5 ± 0.7 on a 5-point Likert scale), with no significant differences between professions (*p* = 0.836). Similarly, e-cigarette recommendations for tobacco product cessation remained low across professions (13%, 85/637), with no significant differences found (*p* = 0.072). The recommendation of e-cigarettes for tobacco cessation is associated with perceived safety, lower respiratory irritation, lower coughing, a lower risk of cancer, and a lower risk for secondhand smokers when compared to traditional cigarettes (*p* < 0.05). *Conclusions*: Most healthcare providers were unsure about the safety of e-cigarettes; about 13% of providers suggested using them for tobacco product cessation, with safety perceptions influencing recommendations.

## 1. Introduction

Electronic cigarettes, also called e-cigarettes or vaping devices, are electric devices that heat a liquid solution and produce an inhalable aerosol. Thailand’s stance on e-cigarettes is driven by public health concerns as well as the risk of nicotine addiction. Additionally, Thailand has made efforts to reduce smoking rates and protect against the potential health risks associated with e-cigarette use. Although e-cigarettes have been prohibited from importation, manufacture, sale, and possession in Thailand, there is currently an upward trend in their illegal use, especially among adolescents [1,2,3].

E-cigarettes were launched on the market with the promise of being less harmful than traditional cigarettes [4], and they contained less nicotine, carbonyl compounds, nitrosamines, and other toxins [5]. Although the evidence of e-cigarettes’ effects on the respiratory and cardiovascular systems, as well as their effects on brain development and cognition and cancer risk, has been published increasingly [4,6,7,8,9,10], there is still controversy about their safety, as harmless as they are claimed to be, especially about their unknown long-term effects [11].

Additionally, studies have discussed the benefits of nicotine-containing e-cigarettes for tobacco product cessation. Some studies have shown that using e-cigarettes for tobacco product cessation is more effective than nicotine replacement therapy and enhances the rate of tobacco product cessation when combined with nicotine patches [12,13]. However, several studies revealed a decreased success rate in quitting smoking with e-cigarettes [14], raising concerns about the possibility of using e-cigarettes as a gateway to other tobacco use [15]. Because there is no consensus on the efficacy of e-cigarettes for tobacco product cessation, they are not officially recommended as a tobacco product cessation therapy [16].

Because smoking is one of the leading causes of preventable illnesses, including respiratory diseases, cardiovascular disease, and cancer, many countries, including Thailand, have implemented tobacco product cessation service programs to provide services and facilitate treatment for smokers who want to quit. The United States Preventive Services Task Force (USPSTF) endorses that all healthcare providers inquire about tobacco usage from adult patients and encourage cessation. They recommend offering behavioral therapies and FDA-approved pharmacological aids to help non-pregnant tobacco users quit. A common approach for healthcare providers to assess patients’ tobacco use is the 5A’s approach [17]. Similarly, Thailand has also established a National Strategy on Tobacco Control Policy using the 5A’s approach [18]. The 5A’s methods include Ask, Advise, Assess, Assist, and Arrange. With these methods, healthcare providers ask about smoking behaviors and offer clear, specific advice on quitting smoking. Then, providers assess the patients’ willingness to quit smoking as well as potential barriers to quitting and assist them in developing a quit plan that includes a quit date, identifying triggers, and dealing with methods to manage cravings. After that, the healthcare providers arrange follow-up appointments to check success, offer continuous support, and change the quit plan as required. All healthcare professionals in Thailand play roles in this service, with various contributions depending on time, frequency, and activities [19]. Moreover, smoking cessation services have been implemented in several Thai community pharmacies, demonstrating their ability to help smokers quit [20].

Although e-cigarettes are prohibited in Thailand, people can still use them through illegal imports. E-cigarettes for tobacco product cessation are recommended in some countries such as the United Kingdom [21,22], while their effectiveness and safety are controversial. In the absence of standardized and evidence-based treatment recommendations, suggestions for using e-cigarettes as a tobacco product cessation aid may be dependent on healthcare providers’ beliefs and perceptions. Because healthcare providers play an important role in providing tobacco product cessation support, it is necessary to assess the perceptions of healthcare providers concerning e-cigarette safety and their use as a type of tobacco product cessation treatment, which may help inform policy decisions about tobacco control and public health, encourage public health awareness campaigns, and discourage their usage.

Therefore, the objectives of this study were to (1) assess healthcare providers’ perceptions on the safety of e-cigarettes and compare between healthcare providers (doctors, pharmacists, and nurses); (2) assess the recommendation using e-cigarettes for tobacco product cessation among healthcare providers and compare among these groups; and (3) assess the relationship between the perception of the safety of e-cigarettes and the recommendation of e-cigarettes to aid tobacco product cessation.

## 2. Materials and Methods

### 2.1. Study Design and Participants

This cross-sectional study was conducted between January and March 2020. The population consisted of healthcare providers from two tertiary hospitals in northern Thailand: University Hospital (hospital 1) with 2054 providers (350 physicians, 5 dentists, 72 pharmacists, and 1627 nurses) and Chiang Mai Provincial General Hospital (hospital 2) with 871 providers (188 physicians, 28 dentists, 46 pharmacists, and 609 nurses). Using Taro Yamane’s formula with a margin of error of 5.0% [23], a sample size of 610 healthcare providers was determined to ensure proportional representation of the hospitals and types of healthcare providers: 335 from hospital 1 (including 57 physicians, 1 dentist, 12 pharmacists, and 265 nurses) and 275 from hospital 2 (including 59 physicians, 9 dentists, 15 pharmacists, and 192 nurses). To account for an expected 30% rate of the possibility that some participants may not fully complete the questionnaire or may not respond at all, the sample size was increased to 793 healthcare providers (435 from hospital 1 and 358 from hospital 2) to ensure a final valid sample size of 610 participants.

### 2.2. Questionnaire Development and Data Collection

A self-administered questionnaire was developed based on the literature review to answer all the objectives of this study. The literature, such as e-cigarettes, tobacco product cessation, and the roles of healthcare providers in tobacco product cessation, was examined. The questionnaire’s face validity and content validity were examined in consultation with the healthcare providers to make sure it was applicable and pertinent to their circumstances. Three experts in the field, including two pharmacists with over ten years of experience teaching about pharmacotherapy for tobacco product cessation and counseling smokers and one doctor who specialized in tobacco product cessation, were responsible for evaluating the content validity of the questions. They used an index called Item–Objective Congruence (IOC), ranging from −1 to +1, with +1 indicating congruence, 0 indicating doubt, and −1 indicating incongruence, to determine whether each question or answer was appropriate and relevant to the study’s goals. Questions with an IOC score of at least 0.5 were retained, while those with an IOC score of less than 0.5 were either modified or removed after consulting with subject matter experts. Next, the questions were tested for clarity and ease of understanding with healthcare providers. To ensure the reliability of the questionnaire used in the study, a Cronbach’s alpha analysis was conducted with a sample of 30 healthcare providers, resulting in a Cronbach’s alpha coefficient of 0.88. This indicates high internal consistency among the items in the questionnaire.

Finally, the self-administered questionnaire comprised three sections. Section 1 is composed of general information: age, gender, 3 types of healthcare providers (doctors [physicians/dentists], pharmacists, nurses), and hospital affiliation. Section 2 included eight items on opinions about the safety of e-cigarettes. These 8 items were as following: 1 “E-cigarettes are safer than traditional cigarettes”; 2 “E-cigarettes cause less irritation to the respiratory tract compared to traditional cigarettes“; 3 “E-cigarettes offer a smoother breath compared to traditional cigarette“; 4 “E-cigarettes are associated with less coughing compared to traditional cigarettes“; 5 “E-cigarettes pose a lower risk of cancer than traditional cigarettes“; 6 “E-cigarettes are less addictive than traditional cigarettes”; 7 “E-cigarettes have less harmful impacts on secondhand smokers than traditional cigarettes“; 8 “The long-term use of e-cigarettes is not harmful to health”. These eight items provide answers with Likert scale ratings of 1 to 5 (1 = strongly disagree, 2 = disagree, 3 = unsure, 4 = agree, 5 = strongly agree). Finally, Section 3 asked about healthcare providers’ recommendation to use e-cigarettes for tobacco product cessation, with the answer “yes” or “no”. 

The cover page explained the study’s purpose, and the healthcare providers were given consent to participate in this study. 

The data collection from healthcare providers was conducted from January to March 2020. The healthcare providers were invited to participate and were informed about this study, and they had time to read the subject information sheet and ask for clarifications before volunteering to be part of the study. Written consent was obtained from all participants prior to completing the anonymous questionnaire, which did not collect any names, surnames, or privacy information to ensure participant confidentiality. All participants who responded to the questionnaire were included.

### 2.3. Statistical Analysis

Participant data were displayed using descriptive statistics, means and standard deviations (SD) for continuous variables, and frequencies and percentages for categorical data. The groups were compared as appropriate using Fisher’s exact test for categorical data and one-way ANOVA for continuous variables. Binary logistic regression was used to look at the link between the eight factors in the opinions about e-cigarettes and the suggestion to use e-cigarettes to stop smoking. The responses were reclassified into two groups: disagree and agree (strongly disagree, disagree, and unsure = disagree; strongly agree and agree = agree). The opinions on safety were considered independent variables, and the advice to use e-cigarettes to stop smoking was the dependent variable. For univariable analysis, the results were reported as a crude odds ratio (OR), and for multivariable analysis, as an adjusted odds ratio (aOR) with a 95% confidence interval (95% CI); the adjusted covariates were age, sex, hospital setting, type of healthcare practitioner, and experiences in offering tobacco product cessation. A variable’s *p*-value of less than 0.05 indicated that it was statistically significant. Data analysis was performed using STATA Software, Version 14.0.

### 2.4. Ethics Consideration

This study protocol was approved by the Research Ethics Committee, Faculty of Medicine, Chiang Mai University, Thailand (Certificate of Approval No. 186/2019, date of approval: 8 July 2019), and the Research Ethics Committee, Nakornping Hospital (Certificate of Approval No. 034/62, date of approval: 31 July 2019) based on the Declaration of Helsinki, ICH GCP.

## 3. Results

### 3.1. Participant Characteristics

We aimed to have at least 610 participants in this study, but increased the target to 793 to account for a 30% rate of questionnaires unsuitable for analysis. Ultimately, we obtained completed questionnaires from 760 participants, including 173 physicians and dentists, 69 pharmacists, and 518 nurses. The majority of participants were female (84.3%) and were between the ages of 20 and 29 years (36.2%). Over half of the study participants were recruited from the University Hospital. Approximately 20% of healthcare providers had prior experience with tobacco product cessation counseling, with the majority having two to five years of practice. Table 1 provides a complete description of the study group’s demographics.

### 3.2. Opinions on Safety of e-Cigarettes

**Figure 1:**
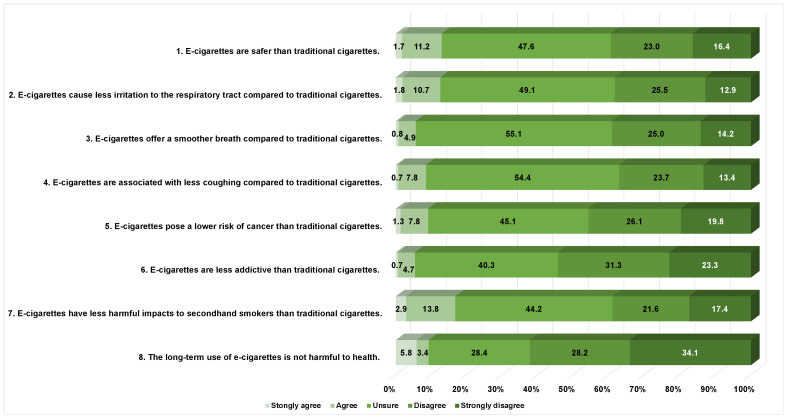
Opinions on safety of e-cigarettes among healthcare providers.

Figure 1 shows the frequency of each opinion, providing a description of the different opinions through survey responses from doctors, pharmacists, and nurses. Healthcare providers were generally unsure about the safety of e-cigarettes. Many providers “disagreed” and “strongly disagreed” that e-cigarettes pose a lower risk of cancer (45.9%), are less addictive (54.6%), and do not cause long-term health problems (62.3%). Table 2 revealed a detailed breakdown of opinions regarding e-cigarettes’ safety among different groups of healthcare providers. There were no significant differences in the means of healthcare providers’ opinions on each item regarding the safety of e-cigarettes among different groups. Supplementary Table S1 provides additional data on the proportion of opinions on e-cigarette safety using five Likert-scale answers.

### 3.3. Recommendation on e-Cigarette Use in Tobacco Product Cessation and Association with Opinions on e-Cigarettes’ Safety

**Figure 2:**
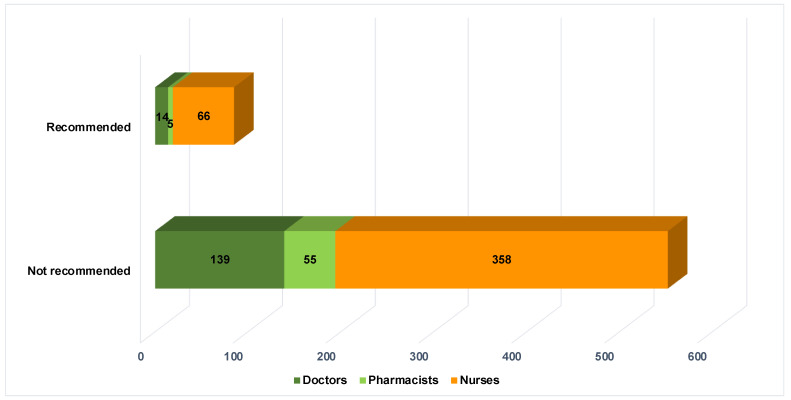
Suggestions on the use of e-cigarettes for tobacco product cessation by types of healthcare providers.

Of the 637 healthcare professionals surveyed, including doctors, pharmacists, and nurses, only 85 (13.3%) recommended using e-cigarettes for tobacco product cessation, as illustrated in Figure 2, which categorized the distribution of suggestions based on the respondents’ professional roles. The suggestions from doctors (9.1%), pharmacists (8.3%), and nurses (15.6%) did not show significant differences (*p*-value = 0.072).

The findings showed that five opinions were significantly associated with recommending e-cigarette use in tobacco product cessation. First, those who agreed that e-cigarettes were safer than traditional cigarettes were more likely to recommend them for tobacco product cessation (aOR = 3.7; 95% CI 2.0–6.7; *p*-value < 0.001) as compared to those who disagreed. Providers who “agree” that e-cigarettes were less irritating to the respiratory tract and cause less coughing than traditional cigarettes were more probable to recommend them for tobacco product cessation than those who “disagree” (aOR = 2.2; 95% CI 1.2–4.1; *p*-value = 0.013 and aOR = 2.6; 95% CI 1.3–5.1; *p*-value = 0.007, respectively). Moreover, with agreement that e-cigarettes pose a lower risk of cancer than traditional cigarettes and have less harmful impacts on secondhand smokers, the healthcare providers were more likely to recommend them for tobacco product cessation (aOR = 2.2; 95%CI 1.1–4.5; *p*-value = 0.030 and aOR = 1.9; 95% CI 1.1–3.4; *p*-value = 0.025, respectively). However, we discovered that the other three opinions—that is, “E-cigarettes provide easier inhalation than traditional cigarettes”, “E-cigarettes are less addictive than traditional cigarettes”, and “The long-term use of e-cigarettes is not harmful to health”—were not significantly associated with the recommendation to use e-cigarettes in order to quit smoking. Table 3 shows a detailed investigation of healthcare providers’ opinions of e-cigarette safety in relation to their recommendations for using e-cigarettes as a tobacco product cessation tool based on 637 replies.

## 4. Discussion

Healthcare providers working in hospitals play important roles in providing tobacco product cessation to patients. Although e-cigarettes are illegal in Thailand [24], their use is increasing, including for quitting smoking. As health concerns about vaping have arisen, the overall efficacy and safety of e-cigarettes have become an ongoing debate in both the public and healthcare professions. This study aimed to contribute knowledge to the field by examining healthcare providers’ opinions on the safety of e-cigarettes, comparing opinions among healthcare providers (doctors, pharmacists, nurses), assessing their recommendations regarding e-cigarettes for tobacco product cessation, and evaluating the associations between opinions on the safety of e-cigarettes and the recommendation of e-cigarettes for tobacco product cessation among healthcare professionals in Thailand.

### 4.1. Opinions of e-Cigarettes’ Safety

In this study, a questionnaire survey of 760 healthcare providers revealed that about 40% disagreed (23% disagreed, 16.4% strongly disagreed) that e-cigarettes are safer than traditional cigarettes. Consistent with the previous study, about 40% of physicians disagreed (27% disagreed, 13.7% totally disagreed) that e-cigarettes are less harmful than traditional cigarettes [25]. The lack of long-term safety data on e-cigarettes is a major concern, as the effects of inhaling these potentially dangerous compounds are unknown. Furthermore, the long-term effects of propylene glycol use in e-cigarettes have not been fully investigated [26,27]. In accordance with this issue, while many of our respondents were unsure about the safety of e-cigarettes, over half of healthcare providers agreed that prolonged use was harmful.

In terms of respiratory system effects, we found that around 40% of healthcare providers disagreed that e-cigarettes are less irritating to the respiratory tract, make breathing easier, and cause less coughing than traditional cigarettes. Previous studies have reported an association between e-cigarette use and respiratory problems [28,29]. Current e-cigarette users who have never smoked traditional cigarettes have an increased risk of developing bronchitis symptoms (daily cough, congestion, or phlegm) [30].

It is widely believed that e-cigarettes may cause cancer [31] and raise concern about secondhand smoke from e-cigarettes. One study revealed that over 80% of healthcare professionals agreed that e-cigarette use increases the risk of cancer, cardiovascular disease, and chronic lung disease. However, they perceived that the risk was lower in comparison with traditional cigarettes [25]. Based on the effects of e-cigarettes attributed to nitrosamines, 4-methylnitrosamino)-1-(3-pyridyl)-1-butanone (NNK) and N-Nitrosonornicotine (NNN), which are potent carcinogens in both humans and mice, there may be a higher risk of cancer [32]. Moreover, whereas the previous study showed that 86.1% and 80.5% of physicians agreed that e-cigarettes are carcinogenic and harmful to people around users, or secondhand smoking, respectively [25], our findings showed that 45.9% and 39.0% agreed with these concerns.

Another concern regarding e-cigarettes is their addictive potential. Most e-cigarettes contain nicotine, just like regular cigarettes, which is a highly addictive substance. Moreover, a study showed that e-cigarettes may have a higher addictive potential than traditional cigarettes [33]. Our finding showed that only about 5% of healthcare providers agreed that e-cigarettes are less addictive than traditional cigarettes; 40% of them were unsure. Similar to this study, another study showed that 20.2% of physicians believed that e-cigarettes have fewer addictive possibilities [25]. 

In short, nearly half of our respondents expressed neutrality (neither agree nor disagree) on the safety of e-cigarettes across all questionnaire topics except for the risk associated with prolonged use. This trend could be attributed to the low awareness of e-cigarettes.

### 4.2. Recommendation to Use e-Cigarettes to Aid Tobacco Product Cessation and Associated with Opinions’ Factors

Healthcare providers’ recommendation using e-cigarettes for tobacco product cessation was quite low in this study (about 13%), and the recommendations for e-cigarettes as a tobacco product cessation aid did not differ significantly among healthcare professions. Similarly, a study among 412 physicians in Poland in 2019 [25], 11.5% agreed that “e-cigarettes should be recommended as a tobacco product cessation tool”. The possible reason why healthcare providers in Thailand are reluctant to recommend using e-cigarettes for tobacco product cessation is that e-cigarettes are illegal in Thailand; therefore, they are not available to use for tobacco product cessation. Furthermore, there is a lack of clear evidence on their safety and efficacy for tobacco product cessation [34]. Unlike traditional smoking cessation aids such as nicotine replacement therapy (NRT), which are regulated and approved by health authorities for tobacco product cessation [24], the evidence on the effectiveness of e-cigarettes in quitting smoking is debatable and needs more research [35,36]. Moreover, few healthcare providers in Thailand know about using e-cigarettes for tobacco product cessation, and they are also unsure about the safety and long-term health effects of e-cigarettes. In short, no consensus exists on the efficacy of e-cigarettes for tobacco product cessation, and they are not officially recommended as tobacco product cessation therapy, leading to the low recommendation rate for tobacco product cessation among healthcare professionals in Thailand.

Our study also investigated the relationship between healthcare providers’ opinions about e-cigarettes’ safety and their likelihood of recommending e-cigarettes for tobacco product cessation. After adjusting for covariate variables, we found five significant associations: healthcare providers who agreed with the statements that ‘e-cigarettes are safer than traditional cigarettes’, ‘e-cigarettes cause less irritation to the respiratory tract compared to traditional cigarettes’, ‘e-cigarettes are associated with less coughing compared to traditional cigarettes’, ‘e-cigarettes pose a lower risk of cancer than traditional cigarettes’, and ‘e-cigarettes have less harmful impacts on secondhand smokers’ were more likely to recommend using e-cigarettes as a tobacco product cessation tool, compared to those who disagreed. Similarly, Ofei-Dodoo et al. [37] found that family physicians who recommended e-cigarettes for tobacco product cessation believed that e-cigarettes pose less risk. Meanwhile, those who were concerned about the safety of e-cigarettes and believed there was insufficient evidence to support their efficacy would not recommend them for tobacco product cessation. The prior study showed that physicians who believed that e-cigarettes could reduce secondhand smoke exposure to patients’ families and friends were more likely to recommend them for quitting smoking (OR 1.45; 95% CI 1.15, 1.83; *p*-value = 0.006) [38]. Moreover, with the perception of a lower risk of cancer compared to conventional cigarette smokers, healthcare providers preferred to recommend e-cigarettes for tobacco product cessation (OR 7.16; 95% CI 3.53–14.55; *p*-value < 0.001) [39].

### 4.3. Public Health Implications

Our study is among the few studies in Thailand to investigate among healthcare providers (doctors, pharmacists, nurses) their opinions on the safety of e-cigarettes, their use for tobacco product cessation, and their associations between opinions and recommendations for tobacco product cessation. This study’s findings have several health implications for healthcare providers and tobacco product cessation efforts. It highlights the importance of healthcare providers’ opinions on the safety of e-cigarettes for quitting smoking. Healthcare providers who believe that e-cigarettes are less safe and effective are less likely to recommend them as one of tobacco product cessation tools. However, the recommendation might be affected by insufficient long-term effects data of e-cigarettes. The providers who were concerned about this issue and needed more sufficient and accurate information about safety and efficacy of e-cigarettes for tobacco product cessation were less likely to recommend them.

Although all pharmacy faculties in Thailand teach pharmacy students smoking cessation and tobacco control in accordance with Thai smoking cessation practice guidelines [40,41], these trainings are also included in other healthcare providers’ programs [42,43,44,45,46], findings revealed that half of healthcare providers were unsure about the safety of e-cigarettes. However, most of them do not recommend these products for tobacco product cessation. Similarly, a recent study by Mittal et al. reported that 62% of healthcare providers lacked knowledge and confidence in counseling patients regarding novel tobacco products, even though more than half had previously received tobacco treatment training [47].

The availability and use of e-cigarettes have increased dramatically worldwide, particularly among adolescents and young adults [48]. Moreover, traditional cigarette users are increasingly turning to e-cigarettes as an option for quitting, although they are aware that the efficacy and safety of these devices for tobacco product cessation are still being debated. According to the report titled “Situation of Tobacco Consumption in Thailand 2024” [49], the national average prevalence of e-cigarette use was recorded at 0.14%. With regard to the northern region of Thailand, where Chiang Mai is situated, the prevalence was observed to be 4.6% among adolescents aged 15 to 24. In the United States, e-cigarettes, along with educated patients, are suggested for traditional cigarette users who do not have success with FDA-approved pharmacotherapy; however, only e-cigarettes approved by the US FDA are recommended [50]. Likewise, a recent study, the COSTED trial, proposed criteria to help in the selection of the most effective vape devices for tobacco cessation programs [51]. This issue poses challenges for healthcare providers to deal with. So far, the benefits of e-cigarettes for tobacco product cessation have been controversial, whereas data on the harm of using them has increased. Our study clearly demonstrated that the decision of healthcare professionals to recommend e-cigarettes for tobacco product cessation depends on their beliefs regarding their safety. Therefore, government authorities should provide information on the harm of e-cigarettes to healthcare professionals, not only to discourage them from recommending e-cigarettes but also to encourage them to suggest that their patients avoid using e-cigarettes.

### 4.4. Strength and Limitations of This Study

We acknowledge some limitations. First, the cross-sectional study design restricts our ability to establish causation or determine the direction of the association between healthcare providers’ opinions of e-cigarettes and their likelihood to recommend them for tobacco product cessation. For investigating causation, a cohort study would be more appropriate. To explore how changing opinions affect recommendations over time, longitudinal research is needed. Second, the use of a self-administered questionnaire may introduce recall bias and the likelihood of socially desirable responses or misinterpretations of questions. Lastly, while limited to healthcare providers from two northern Thai hospitals, this study offers valuable insights into healthcare provider views on e-cigarette safety and smoking cessation across professions. Future research using multicenter sampling and mixed methods could enhance understanding across different regions and improve generalizability.

## 5. Conclusions

In conclusion, this study found that a majority of healthcare providers expressed uncertainty regarding the safety of e-cigarettes. There was a clear link between their perception of e-cigarette safety and their recommendation for tobacco product cessation. Those who believed e-cigarettes were safer than traditional cigarettes were more likely to suggest them as an aid in quitting smoking.

## Figures and Tables

**Table 1:** Characteristics of healthcare providers (n = 760).

Characteristics	Doctors (n = 173) n (%)	Pharmacists (n = 69) n (%)	Nurses (n = 518) n (%)	Total (n = 760) n (%)
Sex				
Male	80 (46.2)	11 (15.9)	28 (5.4)	119 (15.7)
Female	93 (53.8)	58 (84.1)	490 (94.6)	641 (84.3)
Age (years)				
20–29	92 (53.2)	23 (33.3)	159 (30.8)	274 (36.2)
30–39	55 (31.8)	26 (37.7)	105 (20.4)	186 (24.5)
40–49	15 (8.7)	17 (24.6)	131 (25.4)	163 (21.5)
≥50	11 (6.4)	3 (4.4)	121 (23.4)	135 (17.8)
Setting				
University Hospital	113 (65.3)	43 (62.3)	290 (56.0)	446 (58.7)
Provincial Hospital	60 (34.7)	26 (37.7)	228 (44.0)	314 (41.3)
Experiences of tobacco product cessation counseling (n = 751)				
No	109 (63.0)	60 (90.9)	431 (84.2)	600 (79.9)
Yes	64 (37.0)	6 (9.1)	81 (15.8)	151 (20.1)
Years of experience with tobacco product cessation (n = 151)				
<2	14 (21.9)	2 (33.3)	19 (23.5)	35 (23.2)
2–5	39 (60.9)	3 (50.0)	19 (23.5)	61 (40.4)
>6	11 (17.2)	1 (16.7)	43 (53.1)	55 (36.4)

**Table 2:** Opinions on safety of e-cigarettes by types of healthcare providers (n = 760).

Opinions on Safety of E-Cigarettes	Mean ^#^ ± SD	Doctors (n = 173) (Mean ± SD)	Pharmacists (n = 69) (Mean ± SD)	Nurses (n = 518) (Mean ± SD)	*p*-Value *
1. E-cigarettes are safer than traditional cigarettes.	2.6 ± 0.9	2.7 ± 0.9	2.6 ± 0.9	2.6 ± 1.0	0.452
2. E-cigarettes cause less irritation to the respiratory tract compared to traditional cigarettes.	2.6 ± 0.9	2.7 ± 0.8	2.6 ± 0.9	2.6 ± 0.9	0.678
3. E-cigarettes offer a smoother breath compared to traditional cigarettes.	2.5 ± 0.8	2.6 ± 0.7	2.4 ± 0.8	2.5 ± 0.8	0.399
4. E-cigarettes are associated with less coughing compared to traditional cigarettes. (n = 755)	2.6 ± 0.8	2.6 ± 0.8	2.5 ± 0.8	2.6 ± 0.9	0.760
5. E-cigarettes pose a lower risk of cancer than traditional cigarettes. (n = 759)	2.4 ± 0.9	2.5 ± 0.9	2.4 ± 0.9	2.4 ± 0.9	0.509
6. E-cigarettes are less addictive than traditional cigarettes.	2.3 ± 0.9	2.2 ± 0.8	2.2 ± 0.8	2.3 ± 0.9	0.120
7. E-cigarettes have less harmful impacts to secondhand smokers than traditional cigarettes. (n = 758)	2.6 ± 1.0	2.7 ± 1.0	2.8 ± 0.9	2.6 ± 1.0	0.363
8. The long-term use of e-cigarettes is not harmful to health.	2.2 ± 1.1	2.2 ± 1.1	2.1 ± 1.1	2.2 ± 1.1	0.706
Average total score (1–8) (n = 748)	2.5 ± 0.7	2.5 ± 0.6	2.4 ± 0.6	2.5 ± 0.7	0.836

Note: ^#^ Mean was calculated based on Likert scale ratings of 1 to 5, in which 1 = strongly disagree, 2 = disagree, 3 = unsure, 4 = agree, and 5 = strongly agree. * one-way ANOVA.

**Table 3:** Relationship between perceptions of the safety of e-cigarettes and recommendations for e-cigarettes to aid tobacco product cessation (n = 637).

	Recommended (n = 85) n	NOT Recommended (n = 552) n	OR (95% CI)	aOR ^#^ (95% CI)
1. E-cigarettes are safer than traditional cigarettes.				
Agree ^a^	25	53	3.9 (2.3–6.8) **	3.7 (2.0–6.7) **
Disagree ^b^	60	499	1.0	1.0
2. E-cigarettes cause less irritation to the respiratory tract compared to traditional cigarettes.				
Agree ^a^	20	58	2.6 (1.5–4.6) *	2.2 (1.2–4.1) *
Disagree ^b^	65	494	1.0	1.0
3. E-cigarettes offer a smoother breath compared to traditional cigarettes.				
Agree ^a^	9	28	2.2 (1.0–4.9) *	2.2 (1.0–5.1)
Disagree ^b^	76	524	1.0	1.0
4. E-cigarettes are associated with less coughing compared to traditional cigarettes. (n = 632)				
Agree ^a^	14	40	2.5 (1.3–4.8) *	2.6 (1.3–5.1) *
Disagree ^b^	71	507	1.0	1.0
5. E-cigarettes pose a lower risk of cancer than traditional cigarettes. (n = 636)				
Agree ^a^	14	44	2.3 (1.2–4.4) *	2.2 (1.1–4.5) *
Disagree ^b^	71	507	1.0	1.0
6. E-cigarettes are less addictive than traditional cigarettes.				
Agree ^a^	9	23	2.7 (1.2–6.1) *	1.9 (0.8–4.7)
Disagree ^b^	76	529	1.0	1.0
7. E-cigarettes have less harmful impacts to secondhand smokers than traditional cigarettes (n = 635)				
Agree ^a^	23	87	2.0 (1.2–3.4) *	1.9 (1.1–3.4) *
Disagree ^b^	62	463	1.0	1.0
8. The long-term use of e-cigarettes is not harmful to health.				
Agree ^a^	3	54	0.3 (0.1–1.1)	0.3 (0.1–1.2)
Disagree ^b^	82	494	1.0	1.0

OR: odds ratio; aOR: adjusted odds ratio; 95% CI: 95% confidence interval. ^#^ Adjusted with sex, age, setting, types of healthcare provider, and experiences of tobacco product cessation counseling. ^a^ Agree included agree, and strongly agree; ^b^ Disagree included unsure, disagree, and strongly disagree. * *p*-value < 0.05; ** *p*-value < 0.001.

## Data Availability

The data presented in this study are available from the corresponding author on reasonable request.

## Supplementary Materials

The following supporting information can be downloaded at: https://www.mdpi.com/article/10.3390/healthcare12141410/s1, Table S1: Opinions on safety of e-cigarettes (n = 760).
