# Supplementary material for: Electronic Cigarettes and Tobacco Product Cessation: A Survey of Healthcare Providers’ Opinions on Safety and Recommendation
Source: Healthcare (Basel). 2024 Jul 15;12(14):1410. doi: 10.3390/healthcare12141410 (PMC11275567; doi:10.3390/healthcare12141410)
Supplement: Supplementary file 1 [file healthcare-12-01410-s001.zip › healthcare-2998918-supplementary.pdf]

Table S1. Opinions on safety of e-cigarettes (n = 760).

Opinions on safety of e-cigarettes	Mean ± SD	Strongly agree (%)	Agree (%)	Unsure (%)	Disagree (%)	Strongly disagree (%)
1. E-cigarettes are safer than traditional cigarettes.	2.6 ± 0.9	1.7	11.2	47.6	23.0	16.4
2. E-cigarettes cause less irritation to the respiratory tract compared to traditional cigarettes.	2.6 ± 0.9	1.8	10.7	49.1	25.5	12.9
3. E-cigarettes offer a smoother breath compared to traditional cigarettes.	2.5 ± 0.8	0.8	4.9	55.1	25.0	14.2
4. E-cigarettes are associated with less coughing compared to traditional cigarettes. (n = 755)	2.6 ± 0.8	0.7	7.8	54.4	23.7	13.4
5. E-cigarettes pose a lower risk of cancer than traditional cigarettes. (n = 759)	2.4 ± 0.9	1.3	7.8	45.1	26.1	19.8
6. E-cigarettes are less addictive than traditional cigarettes.	2.3 ± 0.9	0.7	4.7	40.3	31.3	23.3
7. E-cigarettes have less harmful impacts to secondhand smokers than traditional cigarettes. (n = 758)	2.6 ± 1.0	2.9	13.8	44.2	21.6	17.4
8. The long-term use of e-cigarettes is not harmful to health.	2.2 ± 1.1	5.8	3.4	28.4	28.2	34.1
